# Disrupted Balance of the Oxidant–Antioxidant System in the Pathophysiology of Female Reproduction: Oxidative Stress and Adverse Pregnancy Outcomes

**DOI:** 10.3390/cimb45100511

**Published:** 2023-10-04

**Authors:** József Gábor Joó, Endre Sulyok, József Bódis, László Kornya

**Affiliations:** 1 Department of Obstetrics and Gynecology, Semmelweis University, 1088 Budapest, Hungary; 2 Faculty of Health Sciences, University of Pécs, 7621 Pécs, Hungary; 3 Central Hospital of South Pest National Institute of Hematology and Infectious Diseases, 1476 Budapest, Hungary

**Keywords:** oxidative stress, reactive oxygen species, antioxidants, placenta, pre-eclampsia, IUGR, premature delivery

## Abstract

The significance of oxidative stress in the pathophysiology of male reproductive processes has been closely studied in the last two decades. Recently, it has become clear that oxidative stress can lead to numerous pathological conditions during female reproductive processes as well, contributing to the development of endometriosis, polycystic ovary syndrome and various forms of infertility. During pregnancy, physiological generation of reactive oxygen species (ROS) occurs in association with several developmental processes including oocyte maturation and implantation. An overproduction of ROS can lead to disturbances in fetal development and increases the risk for missed abortion, intrauterine growth restriction, pre-eclampsia, premature delivery and gestational diabetes. Our review focuses on the etiological role of the disrupted oxidant–antioxidant system during human gestation as it relates to adverse pregnancy outcomes.

## 1. Introduction and Methods

Our review attempts to give a summary of the literature on the etiological role of oxidative stress in the background of pathological conditions in pregnancy. We tried to find the location of disrupted oxidant–antioxidant balance in the complex etiology of miscarriage, hypertensive disorders in pregnancy, intrauterine growth restriction, preterm delivery and gestational mellitus.

The study was conducted according to the Preferred Reporting Items for Reviews (PRISMA) protocol. The study protocol included the following stages: 1. primary research of the systematic literature using search engines and 2. selection of the studies to be included according to our inclusion and exclusion criteria.

We included papers a. from any geographic locations; b. with any publication date; c. written in any language; and d. assessing the relationship between oxidative stress and each physiological and pathological condition discussed in the study. We excluded a. editorials; b. abstracts; c. case–controls; and d. expert opinions.

## 2. Oxidative Stress: Basic Mechanisms

Oxygen is indispensable for the proper functioning of all aerobic biological systems; however, oxygen-related processes also lead to the production of reactive oxygen species (ROS). Several factors including ultraviolet radiation, smoking, alcohol consumption, chronic infections, inflammatory disorders and nonsteroid anti-inflammatory medications (NSAIDs) are known to contribute to this process [1,2]. Under physiological conditions, most cells have the ability to counteract the potential negative effect of ROS production. When this cellular protective ability is diminished and/or ROS production reaches a critical limit, oxidative stress (OS) ensues [1]. Several different mechanisms can lead to ROS production and oxidative stress. This paper describes the general effect of oxidative stress on gestational disorders irrespective of the specific mechanisms involved.

The most important ROS include superoxide anions (O_2_^−^•), hydroxyl radicals (•OH), peroxyl radicals (ROO•), alcoxy radicals (RO•) and hydroxyl peroxyl radicals (HO_2_•) [2]. Although not a free radical, hydrogen-peroxide (H_2_O_2_) is of particularly high importance as it can induce nucleic acid damage through its effect on ROS-like hydroxyl radical production. H_2_O_2_ is formed in vivo through a dismutation reaction catalyzed by superoxide dismutase (SOD). H_2_O_2_ can also induce cellular damage through the inactivation of several key enzymes including glyceraldehyde-3-phosphate-dehydrogenase, which plays an important role in energy-producing intracellular processes [2,3]. During episodes of environmental stress, oxidative damage in cells can increase dramatically, causing damage to their structures. The harmful effect of ROS include damage on DNA and RNA, lipid peroxidation of polyunsaturated fatty acids as well as oxidation of proteins [3].

Reactive nitrogen species are a family of antimicrobial molecules derived from nitric oxide (•NO) and superoxide (O_2_^−^•) produced via the enzymatic activity of nitric oxide synthase 2 (NOS2) and NADPH oxidase, respectively. NOS2 is expressed primarily in macrophages through the effect of cytokines and microbial products [3]. Reactive nitrogen species, together with the ROS, can damage cells, causing nitrosative stress.

In the human body, several antioxidants can protect the organism against the effects of oxidative stress. Under physiological conditions, the human organism has two types of antioxidants: (1) enzymatic antioxidants such as superoxide dismutase (SOD), glutathione peroxidase (GPx), glutathione reductase (GSR) and catalase (CAT) and (2) nonenzymatic antioxidants including primarily diet-related substances such as vitamin C and E, beta carotene, selenium, zinc, taurine, manganese and many others [4].

## 3. Effect of Oxidative Stress on Reproductive Cells

Oxidative stress may influence fertility in both sexes through the suppression of the normal functioning of reproductive cells [5]. Although the scientific literature is quite extensive on the deleterious effects of OS on oocytes, it continues to lag behind in knowledge relating to the interaction between OS and sperm cells. Recent data reveal an essential role of OS in the loss of proliferation capacity of oocytes post ovulation. At this stage, the sensitivity of oocytes to OS surpasses that of the sperm cells. If oxidative stress is present during the first 48–72 h of the post-ovulatory period, ROS induces an apoptotic process in oocytes. It is noteworthy that the contribution of OS to chromosomal nondisjunction was already recognized over a quarter of a century ago with the implication that this effect tends to be more prevalent with increasing maternal age [6].

During meiosis, cohesion between sister chromatids keeps recombinant homologs physically attached and a premature loss of this cohesion can lead to mis-segregation. Extensive evidence suggests that meiotic cohesion deteriorates during oocyte aging. The aging process is accelerated by oxidative damage [6]. The basic mechanism behind the harmful effect of OS on oocytes both in vivo and in vitro also involves mitochondrial release of electrons leading to lipid peroxidation, protein modification and other deleterious biochemical reactions including increased apoptosis in granulosa cells [7].

The clinical use of antioxidants may significantly improve fertility in both sexes by reducing the harmful effects of OS on reproductive cells. However, since the intensity of oxidative stress is highly variable in both the prenatal period and during pregnancy in general, the extent of antioxidant supplementation needs to be carefully adjusted to actual needs. Preconception counseling with close gynecology or andrology follow up may provide a unique opportunity for both antioxidant supplementation and related patient education [8].

## 4. The Physiological Role of ROS in the Female Reproductive Process

**Figure 1:**
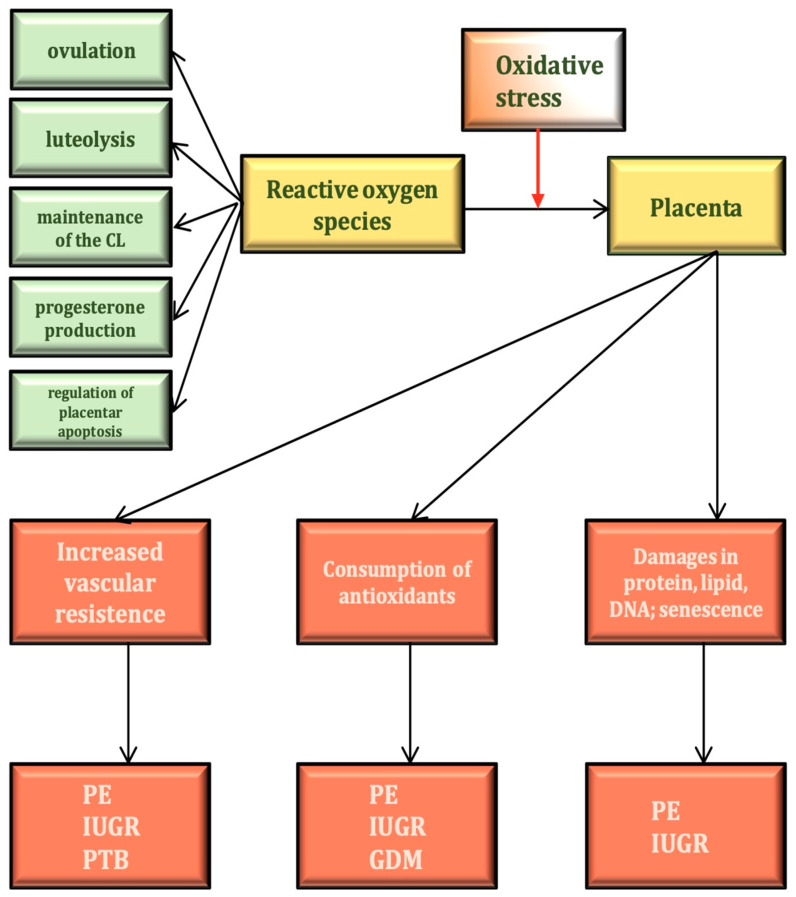
The physiological and pathophysiological effects of ROS in pregnancy. CL: corpus luteum; GDM: gestational diabetes mellitus; IUGR: intrauterine growth restriction; PE: pre-eclampsia; PTB: preterm birth.

The presence of ROS is indispensable for several of the basic functions of the human ovary. These include hormone production, the maturation of the oocyte, ovulation and implantation [9]. The presence of ROS is required for ovulation induction (Figure 1). It is well known that excessive antioxidant effects result in impaired ovulation [10]. During electron transport, electron leakage occurs, which leads to ROS production [11]. Before ovulation, intensive electron transport, enzymatic catalysis of prostanoids as well as free radical reactions are necessary to produce various prostaglandins. Subsequently, the nondominant follicles undergo apoptosis, another ROS-dependent process. ROS-like H_2_O_2_ at lower doses probably induces cell survival responses, whereas at higher doses it activates apoptosis. The p53 protein also plays a key role in the control of cellular stress responses, inducing either cell cycle arrest or apoptosis depending on the context. If oxidative stress increases, p53 induces cell death by downregulating prosurvival genes like Bcl-2, Bcl-XL or survivin [12]. In the corpus luteum, functional luteolysis is also induced by ROS (Table 1).

ROS is also essential for the fusion of oocytes and sperm cells during conception [12] as freshly ejaculated sperm cells cannot fertilize the oocyte until they have spent a certain amount of time in a suitable environment with ROS present. ROS have been reported to promote sperm capacitation with an increase in tyrosine phosphorylation of proteins [12].

Reactive nitrogen species share nitric oxide (NO) as a common progenitor. NO is produced via nitric oxide synthase (NOS) from L-arginine. NOS has three main isoforms: epithelial NOS (eNOS) involved in vascular regulation; neuronal NOS (nNOS) linked to intracellular signaling; and inducible NOS (iNOS), which has several different functions [12].

NO, described as a diffusible radical, is a substance with multiple functions. NO is a key factor in the regulation of the circulatory system, usually causing vasodilation. Evidence also indicates the involvement of NO in meiosis induction. NO also has a basic role in oocyte maturation, fertilization and implantation as well as intrauterine development. Moreover, NO is necessary for the maintenance of oocyte functionality [13]. The iNOS and eNOS isoforms are both linked to ovulation. As NO synthesis increases during follicle development, an increased amount of estrogen is produced. NO also takes part in the regulation of different basic uterine functions. Specifically, NO plays a key role in the regulation of uterine smooth muscle function. In pre-eclampsia, alterations in gene expression for both eNOS and iNOS are also known to occur [13].

## 5. Markers of Oxidative Stress in Obstetrics

An imbalance between pro-oxidant and antioxidant substances often results in oxidative stress that may lead to significant tissue-, organ- or even organ-system-level injury. Beside ROS, reactive nitrogen species also participate in this process, increasing the risk for a wide array of pathological conditions ranging from malignancy, cardiovascular disease, diabetes mellitus and neurodegenerative disorders to diseases of the female reproductive system [13,14,15,16]. Important examples of the latter include polycystic ovary syndrome (PCOS) and endometriosis, conditions that signal organ dysfunction associated with infertility risk. The pathophysiology of the most important obstetric complications including pre-eclampsia (PE), gestational diabetes mellitus (GDM) and intrauterine growth restriction (IUGR) nearly always involves placental OS; in addition, oxidant vs. antioxidant imbalance with oxidant predominance also increases the risk for habitual abortion and premature delivery [17,18,19].

Characterization of this imbalance and consequent OS involves identification of quantitative markers. In recent years, a fairly large number of these have been identified. A recent meta-analysis from 2022 gave a comprehensive description of the significance of OS in gestational disorders, detailing the role of these markers [19]. Due to their large number, we should focus on markers that are most relevant in the pathomechanisms of obstetric disorders.

The latest guidelines propose that SOD activity should be used for the characterization and quantitation of OS risk in general. More specifically, for DNA and RNA damage, 8-hydroxydeoxyguanosine (8-OHdG) is recommended; for lipid peroxidation, malondialdehyde (MDA) is recommended; and for the characterization of OS in gestational disorders nitrogen monoxide (NO), total antioxidant capacity (TAC), total oxidant status (TOS), oxidative stress index (OSI) and glutathione (GSH) are recommended [19,20,21,22]. Total antioxidant capacity denotes the cumulative effect of all antioxidants present in a biological sample, whereas total oxidant status characterizes the cumulative oxidant effects in the whole organism. The oxidative stress index is defined as the ratio of TOS/TAC, describing the balance of oxidative vs. antioxidant effects in the organism.

Specific markers have been identified that predict outcomes in specific obstetric conditions. In pre-eclampsia and associated IUGR, the best predictors for outcomes include MDA, GSH, CA, free thiol (FT), leptin, ischemia-modified albumin (IMA) and receptor for advanced glycation end products (sRAGE) [20,21,22,23,24,25,26]. In gestational diabetes, MDA (malondialdehyde), TAC, GSH, CAT and NO have been recommended as specific predictors [19,27,28,29]. In premature delivery, the presence of several specific markers for OS have been identified [19] as 8-OHdG, GPx, CAT and NO [30,31,32,33,34], as well as TAC, TOS and OSI [35,36,37] (Table 2).

## 6. Regulation of Oxidant–Antioxidant Balance during Physiological Pregnancy

Under physiological conditions, fetal development is strongly dependent on a constant and adequate supply of both nutrients and oxygen. However, both fetal and maternal metabolism naturally involve the production of ROS. Therefore, normal fetal development also depends on the maintenance of a healthy balance between oxidant and antioxidant effects [4]. The maintenance of such a balance is influenced by a sharp increase in the metabolic rate with enhanced oxygen consumption and increased lipid catabolism during the second trimester. Moreover, during the third trimester, insulin resistance and a further increase in lipid metabolism also lead to increased ROS production [18,38]. The primary location of enhanced ROS production during pregnancy is the placental tissue, which is particularly rich in mitochondria.

Disturbances in oxygen or nutrient supply often lead to both specific gestational pathologies and the development of intrauterine growth restriction for the fetus [18,39]. Although placental TAC is known to gradually increase during the second and third trimesters until the 40th gestational week when it reaches pre-gestation levels, it is still reduced during most of gestation. This is associated with decreased serum albumin, serum bilirubin and vitamin E levels, as well as a reduced activity of SOD [40,41,42]. Reduced SOD activity raises total serum cholesterol and low-density lipoprotein (LDL) levels. The rate of lipid peroxidation also tends to increase during pregnancy. Overall, SOD can be regarded as an especially strong marker for OS during the second and third trimesters [43].

Oxidative stress is also known to affect signal transduction during normal reproductive function. Here, mitogen-activated protein kinase (p38-MAPK), Kelch-like ECH-associated protein-nuclear factor erythroid-2 related factor 2 (Keap1-Nrf2), Jun N-terminal kinase (JNK) and forkhead-box O-class (FOXO) have been recognized as the best markers for both changes in signal transduction in general and apoptosis in particular. Furthermore, reduced activity of nuclear factor erythroid-2 related factor 2 (Nrf2) commonly results in fetal DNA damage, and impaired functioning of FOXO interferes with normal implantation and related endometrial changes [44,45,46,47,48,49]. Most of these factors are also associated with apoptotic imbalance associated with several gestational pathological conditions [50]. Specific effects of OS during the first, second and third trimesters are demonstrated in Table 3.

## 7. Oxidative Stress and Epigenetic Effects

Epigenetic inheritance describes biological processes that result in the transmission of traits to the offspring without structural alterations in the DNA sequence. Environmental conditions including endocrine disruptors, obesity, diet, stress, physical activity and smoking may all exert a strong influence on the epigenetic profile. Epigenetic reprogramming is known to occur during aging and in specific pathological conditions, but also during intrauterine fetal development [51,52]. It has been suggested that disturbed fetal programming during intrauterine development as well as epigenetic alterations in the postnatal period due to harmful environmental exposures may result in long-term changes in genetic expression, increasing the risk for the development of several chronic pathological conditions later in life. These latter conditions include ischemic heart disease, type 2 diabetes mellitus, osteoporosis and malignancy [51,52,53].

Several substances potentially causing epigenetic alterations can also cross the placental barrier, influencing the epigenetics of the developing embryo in the first 12 weeks of gestation. The epigenetic profile of primordial germ cells can also be influenced by factors originating from the environment.

Fetal hypoxia is a prominent stress factor increasing the risk for long-term alterations in gene expression, leading to the development of chronic disease [54,55,56,57].

Cardiovascular disease is the most common clinical condition worldwide. Reactive oxygen species are involved in its pathomechanism; on the other hand, ROS also represent central hubs in cellular signaling networks under physiological conditions. Alterations in the epigenetic landscape coupled with increased ROS production may be an important contributor to the development of cardiovascular pathology. This effect is mediated through DNA methylation, histone modification as well as ATP-dependent alterations to the chromatin and non-coding RNA transcripts [52,53].

In children between the ages of 8 and 13 years, blood pressure tends to be higher among those who suffered from IUGR during intrauterine development and have a significant elevation of lipid peroxidation markers [58,59,60,61,62]. During the gestational period, enhanced lipid peroxidation is commonly identified both in the maternal serum and in the placenta when either pre-eclampsia or IUGR is present.

Intrauterine hypoxia resulting in oxidant/antioxidant imbalance seems to also increase vascular reactivity during adulthood, leading to the development of vascular diseases including hypertension [63]. Nevertheless, OS also appears to cause damage in other organs including the liver, pancreas and kidney. In conditions leading to IUGR, epigenetic effects in utero may thus increase the risk for chronic organ dysfunction later in life [64,65,66].

## 8. Oxidative Stress and the Physiology and Pathophysiology of the Placenta

During gestation, the placenta is primarily responsible for adequate materno–fetal oxygen and nutrient supply. The functional development of the placenta is contingent on post-implantation trophoblast invasion with remodeling of the spiral arteries. Under physiological conditions, this is followed by the development of a low-resistance vascular system in the intervillous space [67]. If this process is disturbed at any phase, placental dysfunction will follow, which can frequently result in pre-eclampsia and/or IUGR.

Placental oxygen transport maintains a high local oxygen concentration in the intervillous space that may lead to some degree of oxidative stress even under physiological conditions. However, this is usually compensated for by the development of increased local antioxidant defense mechanisms [68]. During placentation, the interface between the maternal and fetal circulation continues to develop, leading to increased oxygen transport and a higher risk for OS; the latter is the primary trigger for enhanced local production of antioxidants. Antioxidant production in the placenta is primarily performed by cytotrophoblasts and villous stroma cells. If these cells are unable to produce an adequate amount of antioxidants, OS will trigger local lipid peroxidation as well as DNA and protein damage [69,70,71].

The production of ROS and related OS risk tends to grow as gestation progresses. Histological examination of placental samples during the first trimester shows OS-induced vacuolization in syncytiotrophoblasts, diminished villous surface area and a reduction in mitochondrial number. On the other hand, the structures of both cytotrophoblasts and stroma cells remain largely intact [72]. At this phase of pregnancy, antioxidant production in syncytiotrophoblasts increases, while in cytotrophoblasts and stroma cells antioxidant production is unchanged. This means that the maintenance of oxidant/antioxidant balance in the placenta is primarily performed through syncytiotrophoblast adaptation. Without this adaptation, the risk of miscarriage and missed abortion will substantially increase [73].

Placental apoptosis is also induced by oxidative stress, which may result from both extrinsic effects as well as the intrinsic pathway. In either case, altered gene expression in p53, Bax and the Bcl-2 gene family is a key mechanism enhancing apoptotic activity during placental OS [50].

During the second and third trimesters, OS can be associated with several pathological conditions that may eventually result in clinical disease [74]. The primary mechanism behind these is a sustained increase in placental vascular resistance that impairs maternal blood supply to the fetus, leading to ischemia-reperfusion injury (IRI) at the intervillous space. The source of reperfusion injury is the generation of ROS in large part by mitochondria located at cells near the endothelial surface of placental villi. Local ROS production begins within the first moments of reperfusion. These substances are known to be cytotoxic to the surrounding cells [74,75]. Intrauterine hypoxia also promotes angiogenesis in the chorionic villi, a mechanism closely linked to increased placental gene expression of several angiogenic factors [76]. It is important to note that this compensatory response to placental hypoxia takes several weeks to complete [77,78].

Placental OS may induce three different types of compensatory reactions including (1) increased antioxidant production; (2) enhanced angiogenesis; and (3) inhibition of intraplacental apoptosis. Among these reactions, enhanced angiogenesis is the most important. During angiogenesis, growth factors including vascular endothelial growth factor (VEGF) and cytokines stimulate the hypercapillarization of the placental tissue. An inhibition of apoptosis also occurs with increased expression of antiapoptotic genes including the Bcl gene family [67]. While these changes in placental circulation do occur during normal placental development, protecting placental cells and improving placental function and oxygen delivery, they can also be part of a compensatory mechanism. In the latter case, these mechanisms may also increase the risk for both IUGR and death in utero.

The main therapeutic options to help increase placental antioxidant production include the administration of vitamin C, vitamin E and folic acid. ROS is associated with collagen injury to chorioamniotic membranes, potentially leading to a premature rupture of membranes. Vitamin C is involved in collagen metabolism through its antioxidant activity. Vitamin C also plays an important role in the maintenance of amniotic membrane integrity. The antioxidant effect of vitamin E is due to its chain-breaking antioxidant properties and its function as a lipid peroxyl scavenger [79]. Selenium and N-acetylcysteine supplementation may also be useful [80,81,82,83]. Selenium is an efficient antioxidant supporting humoral and cell-mediated immunity. A low selenium level is associated with IUGR and pre-eclampsia. N-acetylcysteine is an antioxidant and an anti-inflammatory agent that is also protective against some of the newborn neurological complications seen in IUGR and preterm delivery pregnancies [81,83]. It is well established that aspirin is also a good therapeutic option to improve placental circulation in the presence of OS.

Pre-eclampsia is a condition of placental vascular origin. After impaired placentation, ischemia-reperfusion injury may develop with increased production of ROS and cytokines, leading to increased risk for placental OS. If the antioxidant defense is inadequate, OS may lead to local inflammation and pre-eclampsia may eventually develop. Aspirin irreversibly acetylates the platelet enzyme cyclooxygenase, modifying the production of different prostaglandins. It also acts as an anti-inflammatory agent, providing further protection against OS [82]. In normal pregnancy, nitric oxide contributes to the regulation of vascular tone necessary for optimal uterine blood flow. Endothelial cells produce NO, which is a strong vasodilator. Increased ROS production suppresses the expression and function of endothelial nitric oxide synthetase, reducing NO production. The altered balance of NO vs. ROS is known to play a critical role in the pathogenesis of pre-eclampsia. Isosorbide dinitrate, glyceryl trinitrate and S-nitrosoglutathione promote NO production and decrease the effects of oxidative stress [84]. Numerous studies are underway to investigate the potential therapeutic role of these substances with the potential to ameliorate OS-induced placental pathology [84,85]. In addition, pomegranate extract is also under investigation as a potential antiapoptotic agent. The presumed mechanism behind its therapeutic effect is the inhibition of apoptosis through the antioxidant action of punicalagin, which may be similar to that of NO [86,87].

## 9. Oxidative Stress and Early Miscarriage

Habitual abortion is defined as three or more consecutive early pregnancy losses. It has a prevalence of 0.5–2% in women of reproductive age. Several pathological conditions are known to contribute to habitual abortion including uterine developmental disorders, fibroids, intrauterine adhesions due to prior surgery, antiphospholipid syndrome, hypothyreosis, polycystic ovary syndrome, hyperprolactinemia, chromosomal abnormalities, advanced maternal age and many others [88,89]. Recently, new evidence has emerged suggesting a possible etiological role for OS in the pathophysiology of habitual abortion [89]. It is now well established that in early pregnancy an increase in the number of polymorphonuclear (PMN) leukocytes in the maternal blood leads to enhanced ROS production and an increased risk for placental OS [90]. PMN leukocytes contain myeloperoxidase, which serves as the main source for ROS before gestational weeks 10–11 in cases where chorionic villi are exposed to hypoxia [90,91,92,93]. An imbalance of oxidants vs. antioxidants in the placenta is recognized as a major factor contributing to first-trimester spontaneous abortions [94]. Malondialdehyde, an end product of lipid peroxidation, has been shown to be one of the best markers of OS in women who develop habitual abortion. Malondialdehyde production was found to be significantly higher in the placental tissue of these subjects, pointing to the importance of placental OS in the context of habitual abortion [95].

In an attempt to prevent habitual abortion, an additional aspect to the traditional diagnosis and treatment of well-known clinical entities is represented by the identification and elimination of pro-oxidant factors including smoking, alcohol consumption, chronic inflammation or infection. Nevertheless, we should keep in mind that even in the absence of such factors early pregnancy inherently carries the risk for the development of placental OS. This fact may give some weight to the argument that early supplementation of antioxidants starting at the time pregnancy is first discovered or possibly even before in the case of planned pregnancy might contribute to the prevention of spontaneous abortion [89].

## 10. Oxidative Stress and Hypertensive Disorders during Pregnancy

Beside gestational hypertension, elevated blood pressure during pregnancy may also be associated with primary hypertension, white-coat hypertension, chronic kidney disease, pre-eclampsia and HELLP syndrome (hemolysis, elevated liver enzymes and low platelet count) [96]. Risk factors for high blood pressure during pregnancy include obesity, advanced maternal age, primiparity, prior diagnosis of hypertension, multiple pregnancy, gestational diabetes and prior gestational hypertension. Although the etiology of most of these conditions remains largely unknown, recent data provide evidence for a significant contribution of oxidative stress in the entire spectrum of hypertensive disorders during pregnancy [97,98].

Pre-eclampsia remains one of the most important pathological conditions giving rise to hypertension during pregnancy. The worldwide prevalence of pre-eclampsia may be as high as 6–8%. The definition of pre-eclampsia is based on blood pressure values that are consistently higher than 140/90 Hgmm on four hourly measurements coupled with proteinuria exceeding 300 mg/day after the 20th week of gestation. Pre-eclampsia has a major impact on perinatal mortality, with global fetal deaths approaching 50,000 to 60,000 per year [99,100].

Pre-eclampsia is a biphasic disease of placental vascular origin. The first phase is characterized by impaired placentation due to inadequate invasion of cytotrophoblast-derived spiral arteries; the second phase is induced by ischemia-reperfusion injury with increased production of ROS and cytokines leading to placental OS [101,102] (Figure 1).

Oxidative stress is also present during normal pregnancy. When placentation is adequate, free radicals serve as second messengers during physiological processes including proliferation, migration and angiogenesis. Since under normal circumstances the antioxidant defense by SOD, GPx, CAT and vitamins C and E will help eliminate these free radicals, the oxidant/antioxidant balance is maintained and OS-related pathology is prevented. On the other hand, if the antioxidant defense is inadequate, OS may lead to local inflammation and pre-eclampsia may eventually develop [103,104].

Inadequate placentation in pre-eclampsia is closely associated with a TNF_α_-related increase in ROS production. The pre-eclamptic placenta is known to create a hypoxic environment where the production of microparticles is stimulated. In this environment, immune-competent cells including neutrophils and dendritic cells are activated. Activated neutrophils in turn produce even more proinflammatory cytokines including TNF_α_. Activated neutrophils also promote oxidative stress through the action of myeloperoxidases such as NADPH oxidase and xanthine oxidase [104]. Thus, the proinflammatory environment in pre-eclampsia creates a vicious cycle of neutrophil recruitment and ROS production, which the placenta may not be adequately equipped to neutralize. In fact, elevated neutrophil count is consistently observed in maternal peripheral circulation in pre-eclampsia, and it is well known that neutrophils represent an essential source of ROS in this condition [105,106,107,108].

NADPH oxidase produces superoxide anions (O_2_^−^•), which in turn inhibit NO production by interfering with the bioactivity of NO synthase. This is particularly important as NO is an essential protagonist in such basic biological processes as endothelium-dependent dilation, VEGF- and angiopoietin-induced angiogenesis and leukocyte adhesion. During gestation, trophoblast invasion and normal placentation are also NO-dependent. In several clinical disease states, NO consumption is increased due to enhanced degradation of endothelial NO synthase (eNOS). In the latter mechanism, OS is known to be an Important contributor [109,110]. In pre-eclampsia, impaired bioactivity of eNOS also results in reduced NO production, leading to vascular dysfunction, a major pathomechanism in this condition [111,112]. Endothelial NO synthesis may also decrease due to the deficiency of L-arginine, its substrate. Several recent studies have investigated the connection between L-arginine metabolism and the onset of pre-eclampsia. In one study, evidence that early-onset and late-onset pre-eclampsia cannot be differentiated based on parameters of L-arginine metabolism alone was presented [113].

In contrast to pre-eclampsia, gestational hypertension does not typically present with proteinuria. However, decreased placental circulation is known to occur, increasing the risk for placental insufficiency and IUGR. In both gestational hypertension and pre-eclampsia, OS is recognized as a major factor. Furthermore, the mechanism of OS-induced pathology seems to be similar in both conditions [114,115]. It appears that renal OS is primarily responsible for the proteinuria present in pre-eclampsia. This mechanism is remarkably absent in gestational hypertension. The main marker for renal OS is 8-oxo-guanine, which correlates with albuminuria not only in pre-eclampsia but also in diabetes mellitus, where it is also a predictor of sudden cardiac death [116]. Pre-eclampsia is frequently associated with IUGR. This is also an OS-related mechanism and is described in further detail in the following section.

## 11. Oxidative Stress and IUGR

Abnormal placentation may lead to the development of IUGR with or without pre-eclampsia. IUGR is defined as fetal weight below the 10th percentile for gestational age [117]. The etiology of IUGR is multifactorial and may include a wide array of conditions from intrauterine infection, fetal developmental disorders, maternal nutritional disorders and medication-related adverse effects all the way to chronic maternal conditions of diverse etiology. Still, the most common associated condition appears to be placental dysfunction [117].

Early IUGR is defined as growth restriction occurring before gestational week 34. Early IUGR is associated with pre-eclampsia in about 40% of cases. IUGR-associated pre-eclampsia tends to run a more severe course, leading to the occasional development of HELLP syndrome [118]. Late IUGR develops after gestational week 35 and is rarely associated with pre-eclampsia. This condition has a more favorable postnatal prognosis compared to early IUGR.

It has been widely recognized that in most cases of IUGR there is inadequate spiral artery invasion, resulting in defective placentation during early pregnancy. Spiral artery invasion is a high-energy process involving cell division and proliferation. Thus, this mechanism is naturally linked to increased cellular metabolism and ROS production [119,120]. Trophoblast invasion of spinal arteries is inherently linked to the risk for placental OS. Inadequate invasion leads to ischemia-reperfusion injury, increasing placental OS and eventually leading to the development of placental dysfunction [121,122]. Placental OS damages lipid structure in cell membranes, protein structure and both mitochondrial and nuclear DNA. These mechanisms are characteristic in most cases of placental dysfunction. The levels of MDA are typically elevated both in the maternal serum and in the placental tissue, which is associated with increased lipid peroxidation during placental OS [123].

In addition to the characteristic signs of tissue damage described above, the placental tissue in IUGR also shows signs of accelerated aging including telomere shortening and reduced or absent telomerase activity [124,125]. The telomere is a region of repetitive DNA sequences at the end of the chromosome, protecting the DNA structure from the effects of potential DNA loss during cell division. The main function of telomerase is the progressive synthesis of telomeric DNA, replenishing potential DNA losses and thereby preventing cellular aging. Telomere-induced aging has specific markers including p21 and p16 that increase in IUGR while the placental gene expression of the antiapoptotic Bcl-2 gene is reduced [126,127,128].

Placental aging is a natural phenomenon caused by placental OS that occurs naturally as a consequence of an ever-increasing oxygen demand by the growing fetus [129,130]. This is the reason why the risk for intrauterine fetal demise reaches its maximum immediately before delivery where fetal oxygen demand is highest; this creates a mandate for close obstetric monitoring during this critical period.

Another important marker for pre-eclampsia-related IUGR is placental growth factor (PlGF). Reduced placental gene expression for PlGF, a placental angiogenic factor, reflects inadequate syncytiotrophoblast function and consequently it can be regarded as a reliable marker for placental dysfunction [131,132].

## 12. Oxidative Stress and Gestational Diabetes Mellitus

Gestational diabetes mellitus (GDM) is one of the most common obstetric complications during human pregnancy. It generally develops during the second trimester and involves both hyperglycemia and hyperinsulinemia with insulin resistance. This condition usually resolves after delivery, but the risk for type 2 diabetes later in life remains elevated. GDM has an increased overall prevalence with advanced maternal age, high BMI and a positive family history. In Europe, the prevalence is about 2–6%, but with significant differences in different countries and regions; in Africa and Asia, the prevalence tends to be even higher [133,134,135,136].

Even under physiological conditions, human gestation is associated with the development of insulin resistance that is compensated for by an approximately 200–250% increase in maternal insulin production during normal pregnancy. Although the pathophysiology of GDM remains largely unknown, it has been assumed that ß-cell dysfunction within the pancreas may play a role, leading to an inadequate compensatory increase in maternal insulin production [137]. Reduced ß-cell function in turn may be due to autoantibodies directed against pancreatic ß-cells or a genetic mutation interfering with normal cellular function [138,139].

An additional mechanism leading to increased GDM risk involves obesity-related chronic inflammation with increased serum levels of cytokines including BMI (body mass index), tumor necrosis factor alpha (TNF_α_) and C-reactive protein (CRP) [140,141]. Placental OS leads to further increases in these inflammatory cytokines, particularly in cases where maternal BMI is high [142].

As in many other obstetric conditions, GDM also features an imbalance between oxidant and antioxidant effects; specifically, an accumulation of hydrogen peroxide (H_2_O_2_), superoxide anions (O_2_^−^•), hydroxyl radicals (•OH) and organic hydroperoxide (ROOH) is commonly observed [143,144]. Increased placental production of PGF_2α_ reflects enhanced lipid peroxidation during GDM pregnancies [144,145]. Elevated placental PGF_2α_ and maternal serum glucose levels commonly occur together, suggesting a direct link between placental lipid peroxidation and maternal serum glucose regulation [143,144]. Another important marker for placental OS in GDM pregnancies is MDA [24]. On the other hand, the levels of several antioxidant factors including SOD and GSH are significantly reduced in GDM [24].

Impaired fetal development is much more common in GDM compared to normal pregnancies. Here, again, placental OS may be a major contributing factor. Among the developmental disorders associated with GDM, impaired cardiovascular development, holoprosencephaly and caudal regression syndrome are particularly common [146]. There are several reports suggesting that fetal macrosomia due to impaired maternal carbohydrate metabolism is associated with reduced antioxidant defense, particularly with decreases in SOD activity during GDM pregnancy [147,148].

An additional factor contributing to increased GDM risk is excessive iron supplementation. In histological studies, evidence for increased placental lipid peroxidation was found in subjects with iron supplementation exceeding 60 mg per day. Signs of placental OS including DNA damage were also observed. Clinical studies also confirmed an increased prevalence of GDM associated with excessive iron supplementation [149,150,151].

In GDM, endothelial-dysfunction-related cardiovascular morbidity is commonly observed [152]. Advanced maternal age is a risk factor for both hypertension and hypertension-associated cardiovascular morbidity during pregnancy. Since GDM is also associated with advanced maternal age, cardiovascular disease is especially common with advanced age, particularly when it exceeds 40.

## 13. Oxidative Stress and Premature Delivery

Premature delivery is defined as birth before the completion of the 37th gestational week. Since in certain cases gestational age cannot be determined with certainty, an alternative definition is sometimes used: birth weight below 2500 g. The worldwide prevalence of premature delivery using WHO data is estimated to be approximately 11–12%, representing around 13–15 million premature births per year [153]. Established risk factors for premature delivery include multiple pregnancy, pre-eclampsia, intrauterine infections, developmental disorders affecting the uterus, fibroids, gingival disease and certain addictions, among many others [154].

One of the main pathomechanisms underlying premature delivery is placental OS. Specifically, ROS-induced DNA damage with telomere shortening promotes accelerated aging in fetal membrane cells and induces an inflammatory process that is directly responsible for the initiation of premature delivery [155]. Both premature activation of the myometrium and premature rupture of membranes are consequences of placental tissue damage and accelerated aging of fetal membrane cells induced by placental OS [156,157]. The most common etiology for placental-OS-associated premature delivery is intrauterine infection or inflammation. Ascending infections of the uterus are indeed diagnosed in most pregnancies ending in premature delivery [158]. As in the case of either pre-eclampsia or IUGR, placental OS seems to cause tissue damage resulting in placental dysfunction, which is the main pathogenic pathway for premature delivery [122].

In premature delivery, the best marker for placental OS is MDA-signaling-increased placental lipid peroxidation. Several recent studies provide evidence that maternal serum MDA levels are significantly elevated in premature delivery vs. normal pregnancy [159,160,161,162].

Urine PGF_2α_ levels are usually higher in premature delivery cases compared to normal pregnancy and are associated with myometrium activation. On the other hand, when premature delivery is induced by premature rupture of the membranes, serum and urinary PGF_2α_ levels are lower. These seemingly contradictory results may be explained by assuming the presence of different placental OS pathways in premature delivery induced by myometrium activation vs. premature rupture of the membranes [163,164].

Carbonylated proteins are also recognized as important predictors for premature delivery. Significantly increased levels of carbonylated proteins are seen both in the maternal serum and in the umbilical blood [165,166].

It is well established that the oxidant/antioxidant imbalance seen in premature delivery does not only result from increased ROS production, but also from impaired antioxidant defense. Catalase (CAT) activity, determined from vaginal smear in premature delivery cases, is significantly reduced, although umbilical blood levels are not significantly different vs. normal pregnancy. Although the data remain inconclusive, CAT dysregulation in premature delivery is a likely candidate for the decreased antioxidant activity observed in this condition [165,167,168].

Another important antioxidant, ceruloplasmin, is also reduced in the maternal serum in premature delivery cases [169]. Other studies showed additional evidence for impaired antioxidant enzyme activity including reduced SOD and thioredoxin reductase function [35,165,170,171].

## 14. Oxidative Stress and Twin Pregnancy

The incidence of multiple pregnancy is on the rise, partly due to the widespread application of assisted-reproduction techniques. Multiple pregnancy is generally considered to be high risk as it is associated with both a variety of fetal developmental disorders and obstetric complications [165]. The most important among these is premature delivery, which also has a significantly higher prevalence in twin pregnancies. Premature birth makes the infant more vulnerable to the effects of oxidative stress including ROS-induced protein and DNA damage. This may also result in tissue- or organ-level damage and may lead to organ dysfunction later in life [172,173]. It is well known that the enzymes participating in antioxidant defense reach their peak activity during the last 2 to 3 weeks before delivery; with premature delivery, the baby is born before the antioxidant defense systems can reach their optimal capacity. [164,173,174]. As can be expected, the umbilical levels of lipid peroxidation markers including 15-F_2t_-isoprostane and MDA are significantly elevated in multiple compared to single pregnancies [172,175]. Although there is little doubt as to the overall significance of reduced antioxidant activity in twin pregnancies, it is generally accepted in the obstetric literature that in twin pregnancies the markers of increased lipid peroxidation are still the best overall predictors of oxidative-stress-related tissue damage.

## 15. Antioxidant Supplementation during Pregnancy

Prevention and treatment of oxidative-stress-related clinical conditions should be a routine part of obstetric practice. Since oxidative stress is known to contribute to the development of most obstetric complications including pre-eclampsia, IUGR and gestational diabetes mellitus, it is essential to strengthen the maternal antioxidant defense [176]. As an additional benefit beside their anti-inflammatory action, antioxidants also have antiapoptotic and antiangiogenic activity. Ideally, the dosage of antioxidant supplementation should follow the actual extent of oxidative stress.

A potential candidate for antioxidant supplementation is curcumin, found in the commonly used spice turmeric. This antioxidant substance has strong anti-inflammatory activity and is a potent cyclooxygenase-2 (COX-2) inhibitor. Turmeric has been used for both the prevention and treatment of pre-eclampsia [177,178,179]. Admittedly, the beneficial effect of curcumin has been suggested based almost exclusively on animal experiments, while human data remain scarce.

Additional candidates for antioxidant supplementation in pre-eclampsia include epigallocatechin gallate (EGCG) and resveratrol (RESV). Both agents potentiate the vasodilative action of nifedipine, contributing to improved blood pressure control [179,180]. Human data on EGCG and RESV supplementation are abundant, yet their use during pregnancy still lacks definitive confirmation [181].

The role of vitamins C and E in the pathogenesis of premature delivery due to premature rupture of the membranes has long been established. It is well known that vitamin C participates in collagen synthesis, and it increases collagen fiber strength through promoting collagen cross-linking. This action is particularly important in premature delivery as premature rupture of the membranes may be the result of inadequate collagen fiber strength [181,182,183,184]. On the other hand, vitamin E acts as a scavenger for lipid peroxyl radicals and it slows the structural changes in the cervix that would eventually lead to premature delivery [185]. Zinc supplementation has also been shown to decrease the prevalence of premature delivery at a dose of 30 mg daily [186,187].

Overall, it seems that antioxidant supplementation has a definite role in obstetric care, but the precise role for individual antioxidants remains to be elucidated, warranting further clinical studies. Even for antioxidant supplements already used in clinical practice, differences in their bioavailability, pharmacokinetic properties, onset and duration of administration relative to differences in maternal metabolism all need to be carefully considered in order to optimize their benefits. With all these factors kept in mind, consensus-based guidelines are urgently needed.

## Figures and Tables

**Table 1:** Beneficial effects of ROS in the female reproductive system.

Zn-Cu SOD ⇑ → promotion of the development of follicles [9,10]
Synthesis of ovulatory steroids → P450 ⇑–ROS ⇑–blood supply–ovulation [1,9,10]
ROS ⇑ → induction of apoptosis in nondominant follicles [11]
FSH ⇑ → E_2_⇑ → CAT and GSH → protection of dominant follicle against apoptosis [10,11]
ROS ⇑ → NF-_kappa_B ⇑ → PGF_2alfa_ → luteolysis [12]
Sperm cell–oocyte fusion → ROS ⇑ → maintenance of normal corpus luteum function [12]
ROS ⇑ → antioxidant effect ⇑ → progesterone production ⇑ [11]
GPx ⇑ → regulation of apoptosis in the placenta [4]
CAT, SOD, GPx in fetal tissues ⇑ defense of fetoplacental tissue against oxidative stress [4,9]

SOD: superoxide dismutase; P450: cytochrome p450; ROS: reactive oxygen species; FSH: follicle stimulating hormone; CAT: catalase; GSH: glutathione; PGF_2α_: prostaglandin 2-alpha; GPx: glutathione peroxidase.

**Table 2:** Quantitative markers of oxidative stress in the background of pathological conditions in pregnancy.

Condition	Quantitative Markers of Oxidative Stress
pre-eclampsia + IUGR	MDA, GSH, CAT, FT leptin, IMA, sRAGE [20,21,22,23,24,25]
gestational diabetes	MDA, TAC, GSH, CAT, NO [19,27,28,29]
premature delivery	8-OHdG, GPx, CAT, NO, TAC, TOS, OSI [30,31,32,33,36,37]

8-OHdG: 8-hydroxydeoxyguanosine; CAT: catalase; FT: free-thiol; GPx: glutathione peroxidase; GSH: glutathione; IMA: ischemia-modified albumin; IUGR: intrauterine growth restriction; MDA: malondialdehyde; NO: nitric oxide; OSI: oxidative stress index; sRAGE: soluble receptor for advanced glycation end products; TAC: total antioxidant capacity; TOS: total oxidant status.

**Table 3:** Oxidative stress effects during different phases of pregnancy [4].

First Trimester	Second Trimester	Third Trimester
Enhanced placental oxygen supply increases OS risk [49]	Rapid increase in oxygen delivery increases OS risk [16]	Strong OS effect with risk for lipid, protein and DNA damage [34]
Trophoblast invasion inducing development of spiral arteries [12]	Altered perfusion of uterine tissue [20,36]	Fetal DNA damage, impaired fetal development [35]
Increased placental arterial resistance reduces uteroplacental flow [12]	Progressive oxidant/antioxidant imbalance [39]	Enhanced placental apoptosis, placental failure [36,37]
Ischemia [23]	Reduced production of antioxidants [36]	
*MISSED ABORTION*, *IUGR*	*IUGR*	*PREMATURE DELIVERY, IUGR, FETAL DEATH IN UTERO*

OS: oxidative stress; IUGR: intrauterine growth restriction.

## Data Availability

Data available in a publicly accessible repository (www.pubmed.gov).
